# Perspectives on Challenges and Opportunities for Interoperability: Findings From Key Informant Interviews With Stakeholders in Ohio

**DOI:** 10.2196/43848

**Published:** 2023-02-24

**Authors:** Daniel M Walker, Willi L Tarver, Pallavi Jonnalagadda, Lorin Ranbom, Eric W Ford, Saurabh Rahurkar

**Affiliations:** 1 Department of Family and Community Medicine College of Medicine The Ohio State University Columbus, OH United States; 2 The Center for the Advancement of Team Science, Analytics, and Systems Thinking College of Medicine The Ohio State University Columbus, OH United States; 3 Department of Internal Medicine College of Medicine The Ohio State University Columbus, OH United States; 4 Government Resource Center College of Medicine The Ohio State University Columbus, OH United States; 5 Department of Healthcare Organization and Policy School of Public Health University of Alabama Birmingham, AL United States; 6 Department of Biomedical Informatics College of Medicine The Ohio State University Columbus, OH United States

**Keywords:** interoperability, health information exchange, health information technology, electronic health record, usability

## Abstract

**Background:**

Interoperability—the exchange and integration of data across the health care system—remains a challenge despite ongoing policy efforts aimed at promoting interoperability.

**Objective:**

This study aimed to identify current challenges and opportunities to advancing interoperability across stakeholders.

**Methods:**

Primary data were collected through qualitative, semistructured interviews with stakeholders (n=24) in Ohio from July to October 2021. Interviewees were sampled using a stratified purposive sample of key informants from 4 representative groups as follows: acute care and children’s hospital leaders, primary care providers, behavioral health providers, and regional health information exchange networks. Interviews focused on key informant perspectives on electronic health record implementation, the alignment of public policy with organizational strategy, interoperability implementation challenges, and opportunities for health information technology. The interviews were transcribed verbatim followed by rigorous qualitative analysis using directed content analysis.

**Results:**

The findings illuminate themes related to challenges and opportunities for interoperability that align with technological (ie, implementation challenges, mismatches in interoperability capabilities across stakeholders, and opportunities to leverage new technology and integrate social determinants of health data), organizational (ie, facilitators of interoperability and strategic alignment of participation in value-based payment programs with interoperability), and environmental (ie, policy) domains.

**Conclusions:**

Interoperability, although technically feasible for most providers, remains challenging for technological, organizational, and environmental reasons. Our findings suggest that the incorporation of end user considerations into health information technology development, implementation, policy, and standard deployment may support interoperability advancement.

## Introduction

### Background

Starting with the Health Information Technology for Economic and Clinical Health (HITECH) Act in 2009, the United States has invested over US$ 36 billion to promote interoperability—the ability of 2 or more systems to exchange and use information [1-3]—through health information exchange (HIE) networks and electronic health records (EHRs). HITECH promoted the adoption and implementation of certified EHRs by providing financial incentives through its “meaningful use” programs and funded grants that helped establish regional HIE networks [4]. Some of these financial incentive programs, such as the state Medicaid Provider Incentive Program (MPIP) were phased out in 2021. However, subsequent legislation such as the Patient Protection and Affordable Care Act reinforced this financing by advancing payment and care delivery models that use risk-based contracts to incentivize quality of care and patient outcomes, such as accountable care organizations, value-based care [5], and patient-centered medical homes [6], which stand to benefit from enhanced electronic data exchange. Other population health policy programs, including Comprehensive Primary Care (CPC) [7], CPC-Plus [8], and Primary Care First [9], further rely on robust data exchange among regional providers. Ongoing federal policy development has continued and enhanced support for meaningful use programs through the Medicare Access and Children’s Health Insurance Program Reauthorization Act of 2015, leading to the Promoting Interoperability Program in 2018.

Recent reports show that 96% of acute care hospitals and 80% of primary care providers (PCPs) have implemented certified EHRs with interoperability capabilities [10,11]. However, actual use of data from an HIE network in clinical care encounters remains low [12]. Researchers have identified several barriers to the use of HIE networks, including poor user interfaces and lack of leadership support [13-15]. Although interoperable health information technology (HIT) is theorized to address these barriers, it continues to elude the health care system [16,17]. For instance, a recent study found that only 45% of all US hospitals engaged in the 4 core elements of interoperability, such as the capability of different EHR systems to find, send, receive, and use or integrate clinical information with one another [16]. Further, rural and smaller hospitals, ambulatory practices, as well as those ineligible for meaningful use incentives (eg, rehabilitation, long-term care, or behavioral health providers [BHPs]) lag behind large, integrated systems in adopting interoperability [18,19].

To promote interoperability, the 21st Century Cures Act (21CCA; 21CCA 2016) mandated the sharing of certain data elements, placed restrictions on information blocking, and promoted the use of application programming interfaces (APIs; eg, Fast Healthcare Interoperability Resources [FHIR]). The 21CCA also established the Trusted Exchange Framework Common Agreement (TEFCA) that provides an infrastructure model and governing approach for HIE networks [20]. However, in the sixth national survey of HIEs, 56% (84/151) of the regional HIEs planned to participate in TEFCA [21], which points to ongoing challenges that continue to limit data exchange. HIEs that intended to participate in TEFCA already had connections with HIEs in other states and participated in at least one national network. Therefore, the decision to participate in TEFCA may be determined by the alignment of HIE processes with existing data sharing rather than convincing HIEs of the benefits of participation [21].

### Purpose

In alignment with policy efforts geared toward the promotion of interoperability, this study aimed to explore provider perspectives on the current state of interoperability challenges. Given the confluence of timing around the conclusion of state MPIPs and the ramp up of 21CCA and TEFCA, questions remain about the progress and remaining challenges related to achieving an interoperable health system. Moreover, previous research on interoperability typically focuses on a single perspective rather than those of multiple stakeholders. This multistakeholder lens is particularly relevant to consider when examining interoperability, as a key goal of this innovation is to connect disparate parts of the health system. Specifically, we conducted semistructured interviews with a stratified sample of key informants, including providers and individuals in leadership positions representing diverse organizations in Ohio, to identify barriers and facilitators to interoperability. The study’s findings add to the body of knowledge about interoperability and may contribute to the efforts of state agencies and federal policy makers, such as the Office of the National Coordinator for Health Information Technology and the Centers for Medicare and Medicaid Services, to advance interoperability. For instance, our findings may provide evidence that supports alignment between the Health Insurance Portability and Accountability Act (HIPAA) with HITECH and 21CCA. Finally, technology vendors may benefit from increased understanding of the end user perspective of their applications to develop user-friendly software.

## Methods

### Study Setting and Design

We used a cross-sectional qualitative design to solicit multistakeholder perspectives on the state of interoperability in Ohio. Ohio provided financial incentives for the adoption of interoperable EHRs for eligible professionals and hospitals through the MPIP. Eligible professionals included those with an active Ohio Medicaid Provider Agreement, such as physicians, optometrists, dentists, certified nurse-midwives, nurse practitioners, and physician assistants practicing in a federally qualified health center or a rural health center led by a physician assistant. Eligible hospitals were also required to have an active Ohio Medicaid Provider Agreement and include acute care hospitals, critical access hospitals, cancer hospitals, and children’s hospitals. Ineligible providers included most behavioral and mental health, long-term care, and home health providers. MPIP operated through 7 incentive cycles, with the final cycle occurring in 2021. On the basis of the 2021 Ohio Medicaid Electronic Health Records Survey for Practices and Hospitals [22], 23.87% (5593/23,435) of the eligible providers and hospitals had received at least one MPIP payment, 96.06% (5280/5593) of the MPIP recipient providers and hospitals had adopted and used an EHR, and 90.73% (16,188/17,842) of those who did not receive an MPIP payment had adopted and used an EHR. Among the 735 ineligible providers, 72.1% (530/735) reported adopting and using an EHR. Epic Systems Corporation is the most prevalent EHR vendor in the state, with 36.80% (2058/5593) of MPIP recipient providers and hospitals and 56.56% (10,092/17,842) of non-MPIP recipient providers and hospitals using an Epic EHR. However, though Epic remained the EHR of choice for group practices (across multiple or single sites) and hospitals, individual practices were more likely to choose other EHR vendors (eg, NextGen and eClinicalWorks). Among the ineligible providers, there was substantial variation in EHR vendors, and CareLogic was the single most prevalent EHR vendor, with 7.8% (57/735) of providers adopting it. Presently, Ohio has 2 large-scale regional HIEs that facilitate electronic exchange of patient data. Almost 31.64% (7416/23,435) of MPIP eligible providers and hospitals had existing arrangements with the regional HIEs to share electronic patient-level clinical data, whereas 21.5% (158/735) of MPIP ineligible providers participated in the regional HIEs.

Data collection and analysis were guided by the technology-organization-environment (TOE) framework [23]. TOE is an organization-level theory that has been applied to explain how the 3 interacting contextual domains (ie, technology-organization-environment) influence a health care organization’s technology-related decision-making [24].

### Ethics Approval

This study was considered to have minimal risk and was approved by the Ohio State University institutional review board (2021B0378).

### Sample Selection

We used a stratified purposive sampling approach to gather diverse perspectives on interoperability based on four representative groups: (1) acute care and children’s hospital leaders, (2) PCPs, (3) BHPs (ie, providers or organizations that provide care for mental health, substance use disorders, stress-related physical symptoms, and life stressors and crises), and (4) regional HIEs (ie, organizations that facilitate information exchange within a network of facilities within a geographic boundary). In addition, both rural and urban subsamples within each of the 3 provider groups were interviewed to ensure geographic representation. We focused on key informants with first-hand perspectives on HIT adoption and its future directions. These key informants were administrative leaders with clinical and nonclinical backgrounds within organizations with decision-making capacity regarding HIT (eg, chief medical information officers, executive directors, chief executive officers, and strategy officers). The study leads received a list of potential key informants from the Ohio Department of Medicaid. Emails were sent to gauge interest in participation; key informants who agreed to participate (20 of 38 organizations invited) were then interviewed. To eliminate any potential conflict of interest, the Ohio Department of Medicaid was not notified of who agreed or refused to participate, and did not participate in any interviews.

### Data Collection

We conducted semistructured interviews with key informants from July to October 2021. The interview guide was developed to ask about EHR implementation in general, the alignment of public policy with organizational strategy, interoperability implementation challenges, and opportunities to improve HIT effectiveness. The interview guide was piloted with administrative leaders (n=2; eg, chief information officer) at an urban hospital in Ohio. This process yielded two versions of the semistructured interview guide for (1) providers (ie, those from hospitals, primary care, and behavioral health) and (2) representatives of HIEs (Multimedia Appendix 1). All interviews were conducted remotely via Zoom and audio recorded.

### Data Analysis

Interviews were transcribed verbatim and analyzed using directed content analysis—an approach that begins with the a priori codes from the TOE framework yet is permissive of emergent themes [25]. The coding team (DMW, WLT, and LP) met weekly to discuss the interviews and preliminary findings throughout the data collection phase. A preliminary codebook was developed by reviewing transcripts and identifying broad themes that emerged from the interview transcripts to organize the data into the 3 TOE domains. Next, to build consensus on the coding guide, the analysis team collectively reviewed common transcripts (n=3) to compare results, refine the codebook, and reconcile any coding discrepancies. The remaining transcripts were divided by organization type for coding. The team continued to compare findings throughout the analysis phase to achieve thematic saturation. The trustworthiness of our findings is ensured by our rigorous and iterative approach to analysis [26]. NVivo software (version 12) was used to support data coding and analysis.

## Results

### Overview

Overall, 24 key informants were interviewed representing 20 distinct organizations: 5 hospitals, 7 PCPs, 6 BHPs, and 2 HIEs. Of the 20 organizations, 15 (75%) were located in urban areas (Table 1).

Below, we report on the key themes and subthemes of the analysis organized by the domains of the TOE framework, including comparing and contrasting those that cut across organizational types as well as those specific to each individual organization.

**Table 1:** Key informant interview sample characteristics^a^.

Organization type	Interviewees (n=24), n (%)	Organizations (n=20), n (%)	Urban area organizations (n=15), n (%)
Acute care and children’s hospital	5 (21)	5 (25)	4 (27)
Primary care provider	7 (29)	7 (35)	5 (33)
Behavioral health provider	10 (42)	6 (30)	4 (27)
Regional health information exchange	2 (8)	2 (10)	2 (13)

^a^Some interviews (n=3) included multiple key informants.

### Technology Domain

#### Overview

Three themes within the technology domain that related to the usability and technological aspects of interoperability were identified: (1) implementation challenges, (2) interoperability capabilities, and (3) opportunities (Table 2).

**Table 2:** Themes and primary subthemes in the technology domain.

Theme and primary subtheme			Representative quote
**Implementation challenges**			
	Maintaining growing number of applications	“There’s been an explosion of health IT [information technology] applications, vendors and products over the past decade, many of whom overlap and functionality intersect in ways that don’t really allow for great interoperability, so just the challenge of sort of how do you meet all the needs, using all the various products out there, and still have a cohesive, reliable, safe experience is a challenge.” [Hospital representative #15]	
	Integrating diverse sources of data into unified medical record	“Our community health centers are on a lot of different EHR [electronic health record] platforms, and those platforms don’t talk to each other, and so the interoperability that we all desire is still not really there. So, we...are utilizing a health population, a population health tool that can sit over any EHR [electronic health record] platform, and that is allowing us to get some of the data aggregated, in spite of the lack of interoperability and communication between different EHRs.” [Primary care provider #22]	
**Interoperability capabilities**			
	Connecting to state-hosted registries and databases (ie, state immunization registry)	“Every hospital has this issue, is that there’s not really a good way to leverage the data being collected by the state vital statistic[s] for our use...We really get no automatic notification that a patient has died. They die elsewhere, and the state finds out because there’s a death certificate somewhere, but our HIM [health information management] department is sort of stuck almost to the point of reading obituaries trying to figure out what patients to mark...It’s really hard, from our perspective to reach out to a family with an appointment reminder about a patient who died, and it’s just, not only is it horrible customer service and patient experience or family experience to do that...I think more connection points with actually the state for some of this basic stuff like birth records and death records and marriage certificates where names are changing and that information’s sitting there, but it seems to be behind this kind of either bureaucratic or policy firewall.” [Hospital representative #15]	
	Exchanging data across the continuum of care	“In our long-term care systems, it would be nice for us to be able to exchange information about those patients, especially with medications, make sure their medications are set up and they know everything that the patient’s on. One thing, too, is provide our providers with information, a little bit more timely from the long-term care facilities to keep them from being readmitted or admitted to the hospital. So that’s something we’d like to be able to do. Another thing would be the merging of medical records for both mental health and their regular healthcare.” [Primary care provider #6]	
	Reliance on regional HIEs^a^ for data exchange	“I see a lot of potential for us to be able to really be part of that health information exchange network, and use our EHR [electronic health record] system to do a lot of that in the background, as opposed to currently what we are doing is we have access to [regional HIE] portal to get the community health record, the information, but that takes staff time. You know, a lot of training, and so it’s not really fully integrated into our EHR [electronic health record], and it’s not fully integrated into our clinical practice processes.” [Behavioral health provider #17]	
**Opportunities**			
	Using new population health software to improve interoperability	“That’s the beauty of it. So, I...mentioned computer vision^b^, so what that’s compared to if you wanted to do that in the past, you would have had to have a pretty labor-intensive interface between the platform and each individual practice’s EHR [electronic health record], right. And that’s a huge level of effort that most people can’t really get to, and that’s why the computer vision piece of that really makes sense. You don’t have to have that, well, it’s still fancy, it’s fancy in a different way, you don’t need a fancy interface, you’re using the computer vision^a^ to match the patients.” [Hospital representative #21]	
	Integrating care coordination programs to improve social needs referrals	“Back to the social determinants...the opportunity to connect to external things like Aunt Bertha or NowPow or Healthify, one of those products that helps kind of do closed loop referral, and whether we’ll do that within Epic...I think those...are helping us kind of reach our goals around reducing health disparities.” [Primary care provider #14]	
	Using FHIR^c^-based applications to advance interoperability	“We are just implementing our FHIR [Fast Healthcare Interoperability Resources] layer right now. What I will tell you is while FHIR [Fast Healthcare Interoperability Resources] is definitely a direction of the future, it is not broadly deployed in the marketplace and not broadly deployed in the workflow or business applications to great extent. But it is, definitely will be an important factor as we move into the future. But it also will not be the silver bullet that everybody’s hoping it was going to be.” [HIE representative #8]	

^a^HIE: health information exchange*.*

^b^Computer vision is a field of technology that enables devices such as smart cameras to acquire, process, analyze, and interpret text, images, and videos.

^c^FHIR: Fast Healthcare Interoperability Resources.

#### Implementation Challenges

For hospitals, a primary concern focused on the growing number and proliferation of application types (eg, EHR, personal health records, HIE, and population health platforms) that contain health information and can be used in clinical encounters. These applications were viewed as difficult and costly to maintain. Similarly, these applications do not use a common data structure and storage format, resulting in too many places for data to be located and for clinicians to search for useful information. The lack of interoperability necessitates that providers leave the EHR to access other applications. As one hospital representative stated as follows:

...It’s one of those things where now you’ve got to subscribe to it [eg, a regional HIE], and it’s another place [eg, application] for you to go to look for more information [eg, patient clinical data]...There’s too many places for data to land and get sent. People just stop looking.

Hospital representative #10

HIE leadership echoed this perspective, adding that a lack of interoperability was an impediment to creating a complete medical record. PCPs noted similar issues related to the number and type of applications needed to overcome gaps in interoperability, such as population health tools (eg, Innovaccer). In contrast, for BHPs, interoperability across applications was not discussed; instead, remarks focused on off-the-shelf EHR systems being misaligned to the specific requirements for BHPs, such as lacking additional protections for substance abuse data.

#### Interoperability Capabilities

Hospitals described advanced interoperability functionality and attributed their advanced capabilities to their EHR vendor rather than the regional HIEs. The regional HIEs were helpful for exchanging continuity of care documents [27] but did not facilitate the integration of information across EHRs. Typically, hospitals only expressed limitations with interoperability functionality as being a function of data recipients’ capacities. For instance, a major concern for hospitals was connecting and being able to integrate EHR data with state-hosted information systems such as the state immunization registry, vital statistics, death certificates, or birth records.

Hospitals and PCPs both noted limitations of exchanging data across the continuum of care, such as with long-term care and BHPs. PCPs additionally noted interoperability challenges with hospitals in their own health systems even when they all used the same EHR vendor, as some vendors are not capable of data exchange in different instances of the same EHR.

BHPs described much more basic data exchange capabilities relative to hospitals and PCPs. For instance, they mentioned that their data exchange is primarily focused on billing. In contrast to hospitals, BHPs mentioned a greater reliance on the regional HIEs for access to health records from other providers and event notifications. The regional HIEs echoed this relationship and discussed how BHPs lag in their interoperability capabilities.

#### Opportunities

Hospitals described opportunities related to new population health platforms that may be able to improve interoperability without the costs associated with interfacing with different EHR systems or HIEs. Both hospitals and PCPs felt that increasing analytic rigor and predictive modeling with artificial intelligence and machine learning would support their population health efforts. API-based data exchange was generally identified as an opportunity to promote interoperability. Hospitals mentioned that the use of applications with this technology will benefit remote patient monitoring and chronic care management. API-based data sharing was also identified as an opportunity to improve interoperability in behavioral health despite the focus on more basic technological opportunities, such as increasing EHR functionality.

The regional HIEs also shared these perspectives on API-based data sharing and were particularly attentive to FHIR APIs for the development and implementation of EHR-integrated applications to advance interoperability. FHIR-based applications were also expected to improve patient access to their medical records. Although most hospitals and PCPs offered patient access to their medical records through patient portals at the time of our study, these were not unified across providers. FHIR-based applications could potentially enable broader patient access through a unified patient portal that collects information from disparate providers. However, the regional HIEs tempered this enthusiasm, recognizing that FHIR-based applications are not currently broadly deployed in existing technology builds.

Hospitals, PCPs, and HIEs also discussed using care coordination programs (eg, Aunt Bertha) that can track referrals to social service agencies that address social determinants of health (SDoH). These providers described collecting SDoH data in discrete data fields but also noted that the lack of standardization of SDoH data fields remains problematic and results in questionable data quality and limited ability to combine data across sources. Efforts to develop SDoH data standards, such as the Gravity Project [28], were raised as opportunities to improve the interoperability of SDoH data.

### Organization Domain

#### Overview

We identified two themes within the organizational domain that affected interoperability: (1) facilitators and (2) strategic alignment (Table 3).

**Table 3:** Themes and primary subthemes in the organization domain.

Themes and primary subthemes		Representative quote
**Facilitators**		
	Relationship with EHR^a^ vendor	“They [EHR vendor] are as interested in making sure that interoperability happens as what we are and so when we start to look at different interfaces that we need to have built, whether it’s to another vendor, like Cerner or eClinicalWorks, then Epic builds that interface, so that it makes it easier on both ends, to make that connection.” [Hospital representative #3]
	Data standards adoption	“I think standards, the general and the meaningful use did quite a bit of pushing this sort of embracing of standards around things like nomenclatures, terminologies that allow for transmission of information. Prior to this we’re pretty much stuck with HL7 [Health Level 7] and custom specifications, but now with just CPT [current procedural technology] or ICD [International Classification of Disease] but, between SNOMED [Systematized Nomenclature of Medicine] and LOINC [Logical Observation Identifiers, Names and Codes] and RxNorm and CVX [vaccine administered] codes for immunizations and it’s gotten a lot better.” [Hospital representative #15]
	Senior leadership support	“The biggest thing [to support data gathering and integration] is the addition of scribes. Adding on that expense of additional manpower to do that data entry for the providers to get them to where they’re comfortable with...what is pertinent to that visit. It may be an ER [emergency room] visit, is it a recent CT [computerized tomography scan], so that way staff isn’t trying to print the last X-amount of things and really all they wanted was one thing, so trying to streamline that to get the physicians the information they need, but having a team around them, to help them put the information back in and alleviate that work from them.” [Primary care provider #16]
**Strategic alignment**		
	Payment program participation impacts technology purchasing decisions	“We are in CPC [Comprehensive Primary Care]-Ohio and CPC+ [Comprehensive Primary Care Plus]. We’re also doing Primary Care First and I have a number of value-based commercial contracts that we deal with as well. We are not an ACO [accountable care organization]. Our new software with our population health software that’s been added to our regular EMR [electronic medical record] should help greatly with that and that’s the reason we did it is because we’re getting into more value-based contracts. I think that’s something that will improve our outcomes for our patients and improve our financial return as well.” [Primary care provider #6]
	Using interoperability to develop cross-sector alignment and stakeholder consensus	“I’d love to see us in the state of Ohio come together at kind of a developer’s conference or something...How can we come together and figure out how to make this work better for Ohio? And I know that sounds really altruistic because everyone’s trying to run a business and all that, but it just seems like there’s so much overlap and you think—I’ll use the example with us: I’m sitting on a mountain of data. Right, and so it just drives me bonkers to hear of a small mom-and-pop startup software company, who has to go out and buy a big giant data warehouse, you know, big giant SQL server and pay licenses and then they contact all the hospitals in the doctor’s office and say, give me all your data, right. And we’re just duplicating these silos. Not too long ago, I was giving a presentation, I said, how many of your hospital systems have a population health strategy? And, of course, 100% of them raised their hand, right. And I said so you’ve invested millions into giant data warehouses to support population health, you know. Right? And they all go, yeah. Like, well so did all the HIEs^b^ [health information exchanges], so did the state of Ohio, you know. ODH [Ohio Department of Health] is trying to, like we’re all we’re all spending—Microsoft and Oracle and all those guys are making money hand over fist. Because we can’t get ourselves organized.” [HIE representative #4]
	Different perspectives on the value of interoperability	“They didn’t see how interoperability would help them take care of their patients any better. And our team even said, ‘Well, you can get lab results like instantaneously.’ ‘Yeah, you know, but I get them a day late. It’s fine.’ ...I think that’s the other challenge is really, is that one example, or is that some X percent of providers in the state of Ohio who don’t see value in that interoperability.” [HIE representative #4]

^a^EHR: electronic health record.

^b^HIE: health information exchange.

#### Facilitators

A consistent subtheme related to the importance of relationships with the EHR vendor to support interoperability emerged. Hospitals placed considerable value on their relationship with Epic and perceived the high concentration of state-wide Epic institutions and the integration between HIEs and Epic as a benefit. Hospitals also mentioned the push for data standards through meaningful use as facilitating interoperability.

PCPs described the important role of senior leadership, who can designate sufficient human resources for tasks that typically increase clinical workload, such as data gathering before appointments. These staff resources can help physicians access and use information from other sources.

#### Strategic Alignment

A critical driver for interoperability among hospitals was their participation in value-based payment programs and population health initiatives. To facilitate the data exchange and integration for care coordination, billing, and reporting required to support these programs, hospitals reported purchasing EHRs for network partners that lacked these advanced capabilities, thus promoting interoperability among their clinical partners. Likewise, hospitals preferred to develop their own in-house population health analytics platforms rather than outsource this function to HIEs.

PCPs also felt that interoperability is central to achieving strategic goals such as population health management, which is integral to participation in alternative payment models (ie, accountable care organizations, CPC-Ohio, CPC-plus, and Primary Care First). They noted the benefits of event notifications made possible by admission, discharge, and transfer feeds that allow PCPs to be notified when their patients have received care in other settings and follow-up accordingly with them to meet their needs.

Conversely, BHPs indicated that they were not participating in value-based purchasing programs to the same degree. However, similar to hospitals and PCPs, their technology investment decisions were guided by their participation in payment programs with sponsors (eg, Health Resources and Services Administration and Substance Abuse and Mental Health Services Administration) that require specific reports, although they may not necessarily aid in interoperability.

The regional HIEs viewed the development of their exchange networks as an opportunity to advocate for cross-sector alignment, particularly as it pertains to streamlining duplicate efforts toward population health management. They remarked that regional HIEs are in a unique position to negotiate partnerships that address the concerns of different stakeholders. Finally, the regional HIEs noted challenges related to some organizations, particularly BHPs, not viewing interoperability as valuable to their organization or aligning with their strategy.

### Environment Domain

Within the environmental domain, a policy theme was identified that focused on how policy may hinder or facilitate interoperability (Table 4).

Hospitals felt that there was room for a greater policy push for managed care plans to initiate value-based payment contracts and distribute incentives to providers. Hospitals also felt the costs of interoperability, such as establishing admission, discharge, and transfer feeds or registry reporting, fall predominantly on hospitals, yet there are no corresponding changes to reimbursement. Similarly, BHPs also desired additional funding to support their adoption of advanced EHR systems.

All providers, including the regional HIEs, also noted the considerable impact of 42 Code of Federal Regulations (CFR) Part 2 (ie, Substance Abuse Confidentiality Regulation) as limiting interoperability and connectivity with BHPs. Despite the general recognition of the policy’s good intentions, it was viewed as negatively impacting clinical care. Some key informants felt that this policy was incongruent with new information blocking rules as part of the 21CCA. The regional HIEs also saw consequences of this policy with respect to responding to public health emergencies such as the opioid epidemic. BHPs, PCPs, and the regional HIEs all advocated for aligning 42 CFR Part 2 with the HIPAA to clarify what is protected and not shareable versus what can be shared for continuity of care.

**Table 4:** Themes and primary subthemes in the environment domain.

Policy	Representative quote
Push insurance plans to participate in value-based payment programs	“The way to actually advance Triple Aim^a^, right, is, is to have these accountable care organizations and really strongly incent the provider organizations to get on board with them. Well, if it’s like pulling teeth to get the Medicaid [insurance plans] to work with us on that, then that’s limited. And so I haven’t seen a lot of folks...in Columbus really encouraging, like, what are they doing to really encourage that those accountable care incentives get down to the provider level. So there’s the CPC+ [Comprehensive Primary Care Plus] program, which is good to a point, because that gives primary care providers some funds upfront to theoretically invest in all this stuff. We’re experiencing now is [Medicaid insurance plans] came on board for [ACO^b^ Network], but none of the other providers are, and they’re coming up with excuses why they don’t want to do it. It’s like, how come someone in Columbus isn’t telling them to get on board with a [ACO Network] program.” [Hospital representative #21]
Increase funding for adoption of interoperable EHR^c^	“Most people don’t realize going into an implementation is how much it will cost you...more of those incentives, I think, would be beneficial even if it’s for individuals moving to a better record that will allow them to do more of the communication between systems and all that. Our current system will never have that capability.” [Behavioral health provider #18]
Substance abuse confidentiality requirements (42 CFR^d^ Part 2) limit interoperability with behavioral health providers	“When you go back to advocacy, the biggest thing we need to do around that in my world again is to align the Part 2 information with more the HIPAA^e^ [Health Insurance Portability and Accountability Act] guidelines, so we can make sure that that information gets out to the primary care providers, gets out to those entities that are trying to support these people. I think that’s one of the biggest roadblocks to taking the next step and helping with the opioid crisis, because again, I think Ohio has done some great work here, but our hands are kind of tied right now, because of the federal rules. And so advocating through the state up to the feds to better align those rules so information can be shared, is probably pretty key.” [HIE^f^ representative #8]
Align 42 CFR Part 2 with HIPAA to clarify what is protected data and what can be shared	“We would love to see movement on the alignment with 42 CFR [Code of Federal Regulations] and HIPAA [Health Insurance Portability and Accountability Act], which would allow sharing without having to always parse out.” [Behavioral health provider #7]
Information blocking continues to exist	“I think the feds really need to step up some of their efforts a little bit, and I’ve been pretty vocal with folks at ONC [Office of the National Coordinator] in DC is data sharing is one part of that information blocking conversation. We’ve got to got to do a better job really supporting that and pushing on EMR [electronic medical record] vendors to send all the data. It makes it very, very challenging to match up data if somebody says, I can’t send you addresses. Well then, that data is almost worthless when you’re talking about trying to track that by zip code and say, hey public health, you’re having an outbreak in zip code 12345. So, if you don’t get addresses from some of the EMRs [electronic medical records], you know, there’s not enough expectation I don’t think even in the HL7 [Health Level 7] standards and some of the others.” [HIE representative #4]
TEFCA^g^ is helpful to establish standards, but could be expanded to promote adoption of FHIR^h^	“And to my point, the national effort, right now, that is looking at expanding the national scope of exchange, it came out of the Cures Act, called TEFCA, the Trusted Exchange and Common Agreement Framework, the standards that they have just pushed out do not include FHIR [Fast Healthcare Interoperability Resources] as a standard...And again, don’t get me wrong, it is going to be the way of the future, and you have to be able to integrate that into your technology stack, but we’ve got a ways to go.” [HIE representative #8]

^a^Triple Aim refers to an approach to optimizing health system performance based on improving population health, enhancing the care experience, and reducing costs [29].

^b^ACO: accountable care organization.

^c^EHR: electronic health record.

^d^CFR: Code of Federal Regulations.

^e^HIPAA: Health Insurance Portability and Accountability Act.

^f^HIE: health information exchange.

^g^TEFCA: Trusted Exchange Framework Common Agreement.

^h^FHIR: Fast Healthcare Interoperability Resources.

The regional HIEs noted some specific concerns, given their experience in the COVID-19 response. First, they described a need for greater enforcement of information-blocking rules. The implications of information blocking were particularly notable during the pandemic when withheld information about addresses prevented HIE from tracking COVID-19 at the local level. Second, the regional HIEs felt that HIPAA guidance on reporting on geographic areas with less than 20,000 patients is both vague and confusing, limiting population health efforts.

Finally, the regional HIEs also mentioned that TEFCA helps to establish standards but did not include any information about FHIR-based standards. They noted that this update would be helpful to promote adoption of FHIR-based applications [30].

## Discussion

### Principal Findings

The near-ubiquitous adoption of certified EHRs over the past decade has resulted in the capture of vast amounts of data across the care continuum. Recent policy efforts such as the 21CCA aim to promote vendor-agnostic integration of external data into the EHRs of all provider types and address information blocking to mitigate challenges. However, questions persist about how well these efforts assist health care organizations in achieving interoperability. Our study examined perspectives from a variety of provider types in Ohio on the state of interoperability. We identified important barriers and facilitators to interoperability among hospitals, PCPs, BHPs, and regional HIEs.

Our findings related to implementation issues within the technology domain suggest that the proliferation of applications that address various use cases promotes capture of rich data.

However, from an end user perspective, this approach can create inefficiencies because of excess information. User interfaces that do not embed multiple discrete applications within the EHR may ultimately create a fragmented medical record, which makes it harder to find relevant information. These types of information silos of patient data can potentially jeopardize patient safety, care quality, and organizational efficiency [31,32]. Fragmented or siloed information may also contribute to provider burnout [33]. Thus, our findings highlight a need for user-centric approaches in technology design and implementation to translate increased information access to use.

Providers did note that technological advances such as computer vision-based population health software (eg, Innovaccer) can help overcome these barriers by pulling data directly into the EHR without requiring back-end integration. Likewise, the growing support for API-based data sharing can further support interoperable information exchange between dissimilar EHR vendors. APIs can extend EHR capabilities [34], although this potential remains unrealized to date. Recent initiatives such as TEFCA that mandate sharing standardized sets of data and promote the use of FHIR APIs are expected to facilitate interoperability in the coming years [30]. Moreover, the API approach has the potential to facilitate population health analytics using machine learning techniques [35].

Within the technology domain, our findings reiterated a gap between hospitals and PCPs at one end of the spectrum and BHPs at the other with respect to their interoperability capabilities. This gap may be a result of most BHPs being ineligible for federal incentive programs [18]. This omission likely not only disincentivizes the adoption of advanced EHRs capable of interoperability among BHPs but also discourages investment and development by EHR vendors of tools designed to meet the needs of BHPs [36].

In addition, across the technology, organization, and environment domains and across provider types, a notable issue that emerged from the key informant interviews was the limitation on interoperability imposed by 42 CFR Part 2, a rule that restricts sharing substance use and behavioral health data. Our findings suggest that despite its intentions, 42 CFR Part 2 effectively operates to prevent, or severely limit, BHPs from participating in exchange. In addition to potential medical errors, this restriction may result in missing data from analytic data sets used by providers, insurers, or researchers [37]. Further, providers expressed concern that this rule may no longer be in alignment with 21CCA information-blocking rules. Moving forward, modifications to 42 CFR Part 2 may be necessary to support further interoperability. Consideration of end user needs and incorporating perspectives of BHPs are essential in any policy changes to carefully consider the need for privacy related to substance use and behavioral health data balanced against the benefits of interoperability [38]. To this effect, the United States Department of Health and Human Services issued a Notice of Proposed Rulemaking in November 2022 [39]. The Notice of Proposed Rulemaking proposes to permit the use and disclosure of Part 2 records based on a single prior signed consent, to expand prohibitions on the use and disclosure of Part 2 records in legal proceedings and to expand patient rights that align with the HIPAA Privacy Rule. The importance of this issue has recently become more visible because of concerns around the protections of reproductive health information following increased abortion restrictions because of the overturning of the *Roe versus Wade* supreme court case [40,41]. The revisions to Part 2, if enacted, would not only help BHPs engage in interoperability but would also provide greater protection for how sensitive health data can be used in legal proceedings.

Interestingly, hospitals attributed their interoperability capabilities more to their relationship with EHR vendors as opposed to the regional HIEs. This finding is likely a consequence of the strong foothold of Epic in Ohio. From an operational and governance standpoint, providers may face fewer barriers to participating in Epic’s Care Everywhere vendor-mediated HIE network. Conversely, participation with a regional HIE may require further effort to establish data exchange policies that require buy-in from multiple stakeholders. Vendor-mediated HIE may address barriers to use, such as the need to leave the EHR to access information. Nonetheless, these vendor-mediated HIEs may create a divide in HIE participation between providers with different vendors [42]. Indeed, as the HITECH funding period drew to a close, the number of state and regional HIEs declined, partly because of the mergers of regional HIEs, funding challenges, and competition from vendor-mediated HIEs [43]. Vendor-mediated and regional HIEs are not necessarily mutually exclusive, although regional HIEs may be more inclusive of a range of provider types. Policies targeted at supporting regional HIEs may be necessary to counteract the market forces of vendor-mediated HIEs and keep interoperability obtainable for nonhospital or hospital-affiliated practices.

Our findings related to strategic alignment may offer useful policy recommendations; provider participation in value-based payment programs plays a critical role in how providers are considering (or not considering) investments in interoperability. To the extent that most of a provider’s patients are beneficiaries of these programs, providers may expand the breadth of their interoperability functionality, such as participation in regional HIEs or use of population health platforms to meet the needs of that particular patient population [44]. Key informants also described the use of social needs referral platforms; however, developing exchange with non-HIPAA–covered social service or community-based organizations can be challenging without properly aligned incentives [45]. In addition to clarifying HIPAA rules around exchange with noncovered entities, the expansion of value-based payment programs may leverage HIT investment. Further, regional HIEs may be well positioned to advocate for cross-sector strategic alignment.

The providers in our study reported capacity-related challenges in interoperability with public health agencies. Chronically underfunded public health systems impeded efficient and timely electronic information exchange during the COVID-19 pandemic [46]. In response, the Centers for Disease Control and Prevention launched the Data Modernization Initiative, resulting in changes to core data sources and facilitating access to electronic case reports and the COVID electronic laboratory reporting that makes test results available. Other initiatives such as TEFCA, through their emphasis on interoperability, are also expected to mitigate challenges, particularly from differing vocabulary standards. Other barriers to interoperability in public health arise from the complex legal and regulatory environment [47]. Even though 21CCA established a legal framework to address information blocking [48], the HIEs participating in our study reported information blocking that prevented tracking cases of COVID-19. Moving forward, it will be critical to monitor the impact of the Data Modernization Initiative and TEFCA on the interoperability of public health data.

### Limitations

We purposely sampled from multiple stakeholders to gain a representative perspective on interoperability. The design focused on breadth across providers rather than depth within a specific provider type or within a single health care organization. Similarly, the study only included Ohio stakeholders, which minimizes variation in the policy environment and may limit generalizability.

The sampling approach was designed to include individuals with decision-making authority with respect to HIT. These perspectives may differ from those of other end users. Finally, owing to time and resource constraints, the interview guide may not have probed all issues relevant to interoperability but was purposefully open-ended to allow participants to discuss topics they deemed important.

### Conclusions

Our findings suggest that despite the ubiquity of data and applications, seamless interoperability into a comprehensive medical record, both within and across providers, remains out of reach. Technological solutions offer promise to overcome these challenges. Likewise, the expansion of value-based payment programs can further incentivize interoperability. Although policy initiatives to expand interoperability existed, they were often misaligned to operational needs and may not be sufficient to overcome market forces. A policy focus toward embracing user-centric design to incorporate end user experience into HIT development may overcome barriers associated with achieving interoperability.

## Appendix

Multimedia Appendix 1 Interview guides.

## Abbreviations

API: application programming interface
BHP: behavioral health provider
CFR: Code of Federal Regulations
CPC: Comprehensive Primary Care
EHR: electronic health record
FHIR: Fast Healthcare Interoperability Resources
HIE: health information exchange
HIPAA: Health Insurance Portability and Accountability Act
HIT: health information technology
HITECH: Health Information Technology for Economic and Clinical Health
MPIP: Medicaid Provider Incentive Program
PCP: primary care provider
SDoH: social determinants of health
TEFCA: Trusted Exchange Framework Common Agreement
21CCA: 21st Century Cures Act
