# Supplementary material for: Perspectives on Challenges and Opportunities for Interoperability: Findings From Key Informant Interviews With Stakeholders in Ohio
Source: JMIR Med Inform. 2023 Feb 24;11:e43848. doi: 10.2196/43848 (PMC10007006; doi:10.2196/43848)
Supplement: Multimedia Appendix 1 [file medinform_v11i1e43848_app1.docx]

**Health Information Technology for Economic and Clinical Health (HITECH)**

**Environmental Scan**

**Provider Interview Guide**

**BACKGROUND**

First, let us thank you for agreeing to participate in our study. My name is ________ and I am working with the Ohio State University on a study sponsored by the Ohio Department of Medicaid. This study aims to identify the improvements that have been made in the adoption, implementation, and utilization of health information technology by Medicaid health care providers in the state of Ohio.

I would like to talk to you about your experience and understand your perspective as a stakeholder within your organization. I am also interested learning how the COVID-19 pandemic may have influenced the role of health information technologies. The information that you provide will be used to help inform opportunities to expand the use of health information technology moving forward. As you learned during the consent process, this discussion is scheduled to last around one hour. Remember whatever you say in this interview will be kept confidential.

**OVERVIEW OF INTERVIEW TOPICS**

In this interview, I will ask you a series of open-ended questions to get your perspectives about several topics. These topics include:

1. Background information about your roles and responsibilities
2. General Perspectives about Health Information Technology
3. Interoperability
4. Consumer Issues
5. Health Information Technology and public health

**INTRODUCTION TO THE INTERVIEW**

We have scheduled the next 60 minutes to discuss these topics with you. Before we begin the discussion, we need to take you through an informed consent process. In particular, let me make sure that you understand that:

1. Your participation is completely voluntary. If you do choose to talk with us, you may leave the interview at any time.
2. We consider this discussion to be confidential. Your participation is confidential in the sense that your name will not be used in any reports or articles.
3. As part of this study, we will write a report summarizing what we learned from these interviews. We will not use your name or any identifying information in the report. This report may be shared with staff at the Ohio Department of Medicaid, the Government Resource Center at The Ohio State University, or staff at the Centers for Medicare and Medicaid Services.
4. We would also like to record the interview for the purposes of data collection for our research. The recording will not be used to identify you in any way.

Do you have any questions about our study or this interview process?

**Provider Interview Guide**

1. ***Roles and responsibilities***
   - - To start, could you tell me your name and your role at [Organization Name]?
       - How long have you been in this position?
       - What is your primary responsibility in this role?
2. ***General Perspectives About Health Information Technology*** *(****When we say Health Information Technology, we are referring to the Electronic Health Record and the applications and services that connect to it.)***

- What are some of the positive impacts of using health information technology at your organization?
- What have been some of the greatest challenges you have experienced adopting, implementing, or using health information technology?
  - Are you concerned about clinician burnout and health information technology?
- What do you see as the future opportunities for health information technology over the next few years for your organization?
  - How has federal and/or state policy, such as financial incentive programs, supported the use of health information technology in your organization?
    - Are there any ways that you think policy could better support the use of health information technology at your organization?
  - How does your organization’s use of health information technology align with value-based payment programs, such as accountable care organizations, CPC+, CPC-Ohio, Medicare bundled payments, or other private payer-based initiatives?
  - What is your perspective about integrating social determinants of health into the EHR?

1. ***Interoperability – (Next, I’d like to briefly discuss your perceptions of interoperability.)***

- How do you define interoperability?
- What has been helpful in supporting your organization’s efforts towards interoperability?
- What have been challenges in achieving interoperability?
- How have community health information exchanges/ regional health information organizations helped your organization achieve interoperability?
- How has your Electronic Health Record vendor helped your organization achieve interoperability?
- Are there specific providers (I.e. behavioral health, post-acute care, long-term care, public health agencies) or types of information that you would like to be able to exchange interoperable health information with, but you currently do not?
  - - Is there anything specific that would be helpful in achieving this exchange?
  - Do you participate in any provider-to-payer data exchange, such as patient disease registries, care coordination programs, ADT event notifications, performance measurement or quality metrics?
    - Do you anticipate any challenges with providing information to the new Managed Care Plans?
  - [For Hospitals] Do you anticipate any challenges complying with new rules to provide real time notification of admissions, discharges, or transfers?
  - [For non-Hospitals] Do you anticipate any challenges with hospitals you work with complying with new rules to provide real time notification of admissions, discharges, or transfers?

1. ***Consumer issues (I’d like to switch gears and learn about your perceptions of issues that are important to consumers)***

- What kinds of health information technologies does your organization offer to patients, such as personal health records or patient portals?
- How do these technologies help your patients?
- What, if any, issues do consumers face with respect to the uptake and use of these technologies?

1. ***Role of health information technology in emerging public health threats (Next, I’d like to discuss ways that health information technology may be involved in your organization’s response to emerging public health threats, such as COVID and the opioid epidemic.)***
   - - How has health information technology, including telehealth, been integrated to support workflow during the pandemic and optimize patient care?
     - Do you see these changes as lasting beyond the COVID pandemic?
     - Were there any barriers or challenges, either within your organization or at the federal or state policy levels, that needed to be addressed?

- Can you tell me about your organization’s experience using the Ohio Automated Rx Reporting System (OARRS)?
  - Do you experience any challenges exchanging data with OARRS?
  - How does your organization use data from OARRS?

1. ***Other things you would like to share?***
   - - Is there anything else you would like to share with us about the adoption and utilization of health information technology at your organization?
2. ***CLOSING***

Thank you very much for your time. Your responses will be very helpful for improving the adoption and use of health information technology.

**Health Information Technology for Economic and Clinical Health (HITECH)**

**Environmental Scan**

**Non-Provider Interview Guide**

**BACKGROUND**

First, let us thank you for agreeing to participate in our study. My name is ________ and I am working with the Ohio State University on a study sponsored by the Ohio Department of Medicaid. This study aims to identify the improvements that have been made in the adoption, implementation, and utilization of health information technology by Medicaid health care providers in the state of Ohio.

I would like to talk to you about your experience and understand your perspective as a stakeholder within your organization. I am also interested learning how the COVID-19 pandemic may have influenced the role of health information technologies. The information that you provide will be used to help inform opportunities to expand the use of health information technology moving forward. This discussion is scheduled to last around one hour. Remember whatever you say in this interview will be kept confidential.

**OVERVIEW OF INTERVIEW TOPICS**

In this interview, I will ask you a series of open-ended questions to get your perspectives about several topics. These topics include:

1. Background information about your roles and responsibilities

2. General Perspectives

4. Consumer Issues

5. Public health

**INTRODUCTION TO THE INTERVIEW**

We have scheduled the next 60 minutes to discuss these topics with you. Before we begin the discussion, we need to take you through an informed consent process. In particular, let me make sure that you understand that:

a. Your participation is completely voluntary. If you do choose to talk with us, you may leave the interview at any time.

b. We consider this discussion to be confidential. Your participation is confidential in the sense that your name will not be used in any reports or articles.

c. We would also like to record the interview for the purposes of data collection for our research. The recording will not be used to identify you in any way.

Do you have any questions about our study or this interview process?

**Non-Provider Interview Guide**

1. ***Roles and responsibilities – (As an introductory question…)***
   - - To start, could you tell me your name and your role at [Organization Name]?
       - How long have you been in this position?
       - What is your primary responsibility in this role?
2. ***[For HIEs Only] General Perspectives About Health Information Exchange***

- Can you briefly describe the architecture of your health information exchange: how does it work?
  - How do providers access information from the HIE (via a portal, in-EHR app, web browser)?
  - Is your HIE a push or pull model?
  - Does your HIE support FHIR?
- What is the business model for your HIE?
  - How do you describe the value your organization provides to participants?
- Can you tell me about how your HIE vendor has helped meet your needs?
- What types of organizations participate in your health information exchange?
  - Do you exchange data with other HIEs in Ohio or other states?
  - Is data blocking a concern for your HIE?
- Are there any providers (I.e. behavioral health, post-acute care, long-term care, public health agencies) or types of information that currently do not participate in your health information exchange?
  - How, if at all, is your organization working to with these providers to support their participation?
- Can you tell me your ability to exchange data with the Medicaid and commercial managed care plans?
  - Do you send and receive data to the Medicaid and commercial managed care plans? What types of data?
  - Do you experience any challenges with this exchange?
- Are there any social service or community-based organizations that participate in your HIE?
  - How, if at all, is your organization working with these types of organizations to support their participation?
- What is your organization’s long-term vision for your HIE?
  - What stage in development is your organization in realizing this vision?
  - Is the current interoperability technology capable of supporting this vision?
  - What barriers does your organization face in realizing this vision?
- What do you see as the greatest opportunities for your organization?
- Do you receive data from providers in a common framework, such as HL7 C-CDA document architecture?
  - What framework do you use?
  - Are there any specific clinical areas that you receive additional information for, such as maternity care?
- Have you been able to establish Application Programming Interfaces (APIs) for third party entities to retrieve your data?
  - Do you have any examples of how the APIs have been used?
  - Do you have any concerns about having the APIs?
- What role do you see for the Ohio Department of Medicaid in supporting exchange of information?

- How does your organization’s use of health information technology align with value-based payment programs, such as accountable care organizations, CPC+, CPC-Ohio, Medicare bundled payments, or other private payer-based initiatives?

1. ***Consumer issues (I’d like to switch gears and learn about your perceptions of issues that are important to consumers)***

- How does your organization help consumers (i.e. patients) access their health information electronically?
- What, if any, issues do consumers face with respect to the uptake and use of these technologies?

1. ***Role of health information technology in emerging public health threats (Next, I’d like to discuss ways that health information technology may be involved in your organization’s response to emerging public health threats, such as COVID and the opioid epidemic…)***
   - - How has your organization used health information technology, including telehealth, during the pandemic to optimize patient care?
     - Do you see these changes as lasting beyond the COVID pandemic?
     - Were there any barriers or challenges, either within your organization or at the federal or state policy levels, that needed to be addressed?

- How does your organization use health information technology to help address substance abuse issues?
- Can you tell me about any relationship between your organization and the Ohio Automated Rx Reporting System (OARRS)?
  - [If no relationship] Is a relationship with the OARRS system something your HIE considered?
  - [If yes] Do you experience any challenges exchanging data with OARRS?
    - How does your organization use information from OARRS?

1. ***Other things you would like to share?***
   - - Is there anything else you would like to share with us about the use of health information technology in Ohio?
2. ***CLOSING***

Thank you very much for your time. Your responses will be very helpful for improving the adoption and use of health information technology.
